# Primary Tumor‐Associated Loss of the Y Chromosome and Clinical Outcome in Metastatic Colorectal Cancer

**DOI:** 10.1002/gcc.70147

**Published:** 2026-06-14

**Authors:** Anaëlle Isnard, Marie Decraecker, Benjamin Fernandez, Denis Smith, Pierre Dubus, Sandrine Dabernat, Olivier Mansier, Samuel Amintas

**Affiliations:** ^1^ CHU Bordeaux, Tumor Biology and Tumor Bank Laboratory Pessac France; ^2^ BRIC (BoRdeaux Institute of Oncology), INSERM, University of Bordeaux Bordeaux France; ^3^ Oncology Unit Haut Lévêque Hospital, University Hospital Center of Bordeaux Pessac France; ^4^ CHU Bordeaux Colorectal Digestive Surgery Department Bordeaux France; ^5^ CHU Bordeaux, Biochemistry Laboratory Bordeaux France; ^6^ CHU Bordeaux, Hematology Laboratory Bordeaux France; ^7^ Biology of Cardiovascular Diseases INSERM, University of Bordeaux Pessac France

**Keywords:** biomarker, chromosomal instability, colorectal cancer, *KRAS*, loss of Y chromosome, survival

## Abstract

**Background:**

Alterations of the Y chromosome are frequent events in solid tumors, yet their biological and clinical significance remains incompletely understood. In colorectal cancer (CRC), recent studies have suggested context‐dependent roles of Y‐linked genes in tumor progression, while the impact of tumor‐associated loss of the Y chromosome (LoY) in metastatic disease has not been clearly established.

**Methods:**

We retrospectively analyzed primary tumor samples from 91 male patients with metastatic CRC treated at a single institution. Tumor LoY status was assessed using a droplet digital PCR–based assay. Associations between LoY, clinicopathological characteristics, *KRAS/BRAF* mutation status, and patient outcomes, including progression‐free survival (PFS) and overall survival (OS), were evaluated. Exploratory analyses of TCGA‐COAD/READ data were performed using chromosome Y copy‐number segment data, mutation data, and survival information.

**Results:**

Tumor LoY was detected in 46 cases (50.5%) and was more frequent in rectal cancers (*p* = 0.038). LoY was not associated with age, microsatellite instability status, *KRAS* or *BRAF* mutations. While PFS did not differ according to LoY status, OS was longer in patients with LoY‐positive tumors (*p* = 0.046). In multivariable analysis, LoY showed a non‐significant trend toward improved OS (HR = 0.52, 95% CI: 0.25–1.10; *p* = 0.07). In exploratory TCGA analyses, chromosome Y copy‐number signal did not significantly differ between colon and rectal cancers, but lower chromosome Y signal was associated with worse OS, particularly in TCGA‐COAD after adjustment for age and *KRAS*/*BRAF* status.

**Conclusions:**

LoY is a frequent chromosomal alteration in metastatic CRC and appears enriched in rectal primary tumors in our cohort. Its association with clinical outcome may depend on disease stage, molecular background, and analytical methodology. These findings support further investigation of Y chromosome loss as a context‐dependent biomarker in CRC.

## 1 Introduction

Sex differences between males and females can be detected early in life. They are also present later, even to a much greater extent, affecting adulthood across a wide spectrum of biological and phenotypic characteristics [1]. Sex‐related biological differences are linked to multiple parameters, mainly hormonal influences, and sex chromosome–linked genetic factors. Beyond hormonal influences, sex chromosomes themselves represent a major source of genetic asymmetry between males and females and can contribute to cancer‐associated genomic instability.

In the context of cancer, it is well known that sex differences affect multiple parameters, from incidence to patients' outcomes [2]. Cancer incidence is generally higher in men than in women [3], including colorectal cancer [4]. Although this difference has long been attributed to more risky behaviors, such as smoking and alcohol consumption [5], it can persist even after adjustment for these variables. Additionally, multiple studies describe better outcomes for women than for men [6, 7]. These observations tend to indicate that sex‐specific biological mechanisms may contribute to the increased risk and severity of cancers in men, highlighting a potential key biological factor for better understanding the disparities observed in oncology [8].

Interestingly, different studies described loss of the Y chromosome (LoY), a male‐specific genetic attribute, as a genetic event that could influence tumor biology and cancer patients' outcomes. The Y chromosome is well known for its possible loss in leukocytes during aging, being detected in 8%–37% of men after 70 years [9, 10]. In 2014, *Fosberg et al.* reported for the first time in men with LoY in peripheral blood cells an increased risk of non‐hematological cancer. The presence of LoY in peripheral blood also appeared to be linked to an increased all‐cause mortality, as well as non‐hematological cancer‐related deaths [11]. More recently, a group reported a significantly lower Y/X ratio in the circulating blood of colorectal cancer (CRC) patients, compared to healthy patients [12]. These results suggested the higher prevalence of mosaic LoY in CRC patients, questioning its contribution to tumoral processes and cancer biology. However, in this study, the authors only searched for LoY in peripheral white blood cells, without looking at a possible LoY at the tumor tissue level. Importantly, LoY chromosome in peripheral blood cells reflects age‐related mosaicism, whereas tumor‐associated LoY represents a somatic chromosomal alteration arising within cancer cells and potentially linked to clonal evolution. Based on cancer genomic databases, a recent in‐depth global analysis revealed that LoY was frequently detected in solid tumors, representing the most frequently deleted chromosomes in human cancers [13]. At the tumor level, LoY can occur in the context of global genomic instability, appearing as a passenger event, in particular due to the Y chromosome's small size and low genetic content [14] This is in line with the high frequency of LoY in *TP53*‐mutant tumors, which are usually aneuploid and harbor high genomic instability [14]. Conversely, LoY appears to play a driving role in some tumors, such as uveal melanoma, where it represents an early somatic genetic event that strongly predicts poor survival [14]. Additionally, the presence of LoY in tumors has been associated with a worse prognosis in other tumor types like bladder cancer [15]. Although some conflicting reports exist, the presence of an oncogenic *KRAS* mutation has been repeatedly identified as a negative prognostic factor in colorectal cancer (CRC), particularly in the metastatic setting, influencing both treatment response—especially to anti‐EGFR therapy [16], and patient outcomes [17]. Interestingly, LoY seems to be associated with a favorable prognosis in CRC. A recent study investigated the role of *KDM5D*, a gene located on the Y chromosome, in the mechanisms of CRC metastasis and immune‐system escape. The authors reported that the activation of the *KDM5D* gene, driven by *KRAS* oncogenic mutation, led to epigenetic alterations ultimately increasing cancer cell adhesion properties, cell invasiveness, and immune system inhibition [18]. In accordance with these results, LoY and the subsequent *KDM5D* gene deletion were associated with opposite effects, ultimately conferring CRC a better prognosis/more favorable course. While these data support a functional role of Y‐linked genes in colorectal cancer progression, the extent to which tumor LoY itself directly contributes to clinical outcome remains to be clarified. In addition, the prevalence and clinical significance of chromosomal LoY assessed in primary colorectal tumors, particularly in the metastatic setting, remain poorly characterized.

In this context, we investigated in a monocentric cohort of patients with metastatic colorectal cancer (mCRC) the prevalence of primary tumor LoY, its relationship with the *KRAS/BRAF* mutation status, as well as their impact on progression‐free survival (PFS) and overall survival (OS).

## 2 Material and Methods

### 2.1 Patients' Inclusion and Follow‐Up

We conducted a monocentric, retrospective study on male patients treated at Bordeaux University Hospital for metastatic (synchronous or metachronous) colon or rectal adenocarcinoma between January 2019 and December 2021. Clinical, biological, and pathological data were retrospectively collected for all patients at the time of metastatic disease diagnosis from medical records. The following variables were recorded: patient age (in years), anatomical site of the primary tumor (right colon, transverse colon, left colon, or rectum), and the stage of the primary tumor at diagnosis (localized, locally advanced, or metastatic). The date of metastatic disease diagnosis was noted, along with the number of metastatic sites, the presence of liver metastases, and lymph node involvement. Performance status at the time of metastatic diagnosis was assessed using the World Health Organization (WHO) performance scale. Anthropometric measurements included height (cm), weight (kg), and body mass index (BMI, kg/m^2^). Serum levels of carcinoembryonic antigen (CEA, ng/mL) and carbohydrate antigen 19‐9 (CA19‐9, IU/mL) were also recorded. Molecular characteristics of the tumor included *KRAS* and *BRAF* mutational status, as well as microsatellite instability (MSI) status. Data on first‐line treatment for metastatic disease and any therapeutic adjustments made thereafter were also collected. Chemotherapy was administered according to standard regimens. Follow‐up data included the date of last contact and date of death, if applicable. All patients had a minimum follow‐up time of 6 months. This work was performed following the human and ethical principles of research outlined in the Helsinki guidelines and following local statutory requirements. It was accepted by the Bordeaux University Hospital ethics review board on 14/01/2025 (reference CER‐BDX 2025‐005).

### 2.2 Loss of Chromosome Y Analysis

DNA was extracted from a 1 mm punch made in the paraffin block using a Maxwell RSC DNA FFPE Kit (Promega, Charbonnières‐les‐Bains, France). Extracted DNA was eluted in 70 μL of “nuclease‐free” water, and DNA concentration was determined by fluorimetry with the DS11FX automated system (DeNovix). The detection of LoY was performed thanks to an in‐house droplet digital PCR technique as previously described [10]. The primers and probe design used are detailed in Supplemental Table S1. Briefly, 75 ng of DNA was mixed with ddPCR supermix (without dUTP, Biorad), primers (0.9 μM each), and probes (0.25 μM each). The emulsion was generated using the QX‐100 (Biorad), and the number of droplets positive for *AMELX* and *AMELY* was determined on the QX‐200 droplet reader (Biorad) using Quantasoft version 1.5 software (Biorad). At least 10 000 droplets were analyzed in each well. The results were presented as the R ratio, defined as the ratio of the Y chromosome copy number to the X chromosome copy number. We first determined the background noise of our technique by analyzing tissues from 30 control subjects, with DNA extracted from normal colorectal tissue of young (age < 40 years) healthy men, assessed by pathological analysis. The mean R ratio of the cohort was 0.86 with a standard deviation of 0.11. We next evaluated the Y/X ratio in tumor cells using the following formula: *R*′ = $\frac{R-\left(0.86\times \mathrm{TS}\right)}{\mathrm{TT}}$, with “R” corresponding to the ratio between the number of copies of the Y chromosome and the number of copies of the X chromosome obtained in the DNA sample, “TS” and “TT” correspond respectively to the ratio of healthy cells and tumor cells in the tissue sample. This calculation was used to account for both the tumor cellularity and the background noise observed in healthy tissue. Given the background noise, we set our threshold for confirming the presence of the LoY gene at *R* = 0.55 (0.86 − 3 × 0.11). Based on the R ratio, patients were classified into two groups: those with detectable total or partial loss of the Y chromosome (R' < 0.55, LoY group) and those without detectable loss of the Y chromosome (*R*′ > 0.55, Y preserved group).

### 2.3 Outcomes

The primary endpoint of this study was median overall survival (mOS) according to LoY status. Secondary endpoints included median progression‐free survival (mPFS) and the comparison of clinicopathological characteristics stratified by LoY status. mPFS was defined as the time from the diagnosis of metastases to documented disease progression. mOS was defined as the time from the diagnosis of CRC metastases to death from any cause.

### 2.4 TCGA Exploratory Analyses

An exploratory analysis was performed using publicly available TCGA‐COAD and TCGA‐READ data retrieved from the Genomic Data Commons using the TCGAbiolinks R package. Clinical data and unmasked copy‐number segment files were downloaded for both projects. Only male patients with primary tumor samples and available chromosome Y copy‐number segmentation data were included. Masked copy‐number segment files were not used, as these files do not retain chromosome Y information. For each tumor sample, chromosome Y segments were extracted from the unmasked copy‐number segment files. A chromosome Y copy‐number score was calculated as the length‐weighted mean of segment mean values across chromosome Y, using segment size as weight. This score was used as a continuous surrogate of chromosome Y copy‐number signal. Colon and rectal cancers were defined according to the TCGA project of origin, with TCGA‐COAD classified as colon cancer and TCGA‐READ as rectal cancer. The chromosome Y copy‐number score was compared between colon and rectal tumors using the Wilcoxon rank‐sum test. Because chromosome Y copy‐number segment values appeared globally shifted in TCGA male samples, no absolute binary threshold was used to define LoY in the primary analysis. Instead, sensitivity analyses were performed by classifying tumors within the lowest quartile and lowest decile of chromosome Y copy‐number score as having the lowest chromosome Y signal. The proportion of tumors in these categories was compared between colon and rectal cancers using Fisher's exact test. Overall survival was further explored in TCGA male patients with available clinical follow‐up data. Chromosome Y loss was analyzed both as a continuous chromosome Y loss score, defined as the negative value of the length‐weighted mean chromosome Y segment score, and as a categorical variable corresponding to the lowest quartile of chromosome Y copy‐number score. In the combined TCGA‐COAD/READ cohort, Cox proportional hazards models were adjusted for age and tumor location. To account for major molecular prognostic covariates while avoiding overfitting in the smaller rectal cancer subset, additional multivariable survival analyses were performed in TCGA‐COAD only, with adjustment for age, *KRAS* mutation status, and *BRAF* mutation status. The proportional hazards assumption was assessed using Schoenfeld residuals.

### 2.5 Statistical Analysis

For quantitative parameters, a non‐parametric Mann–Whitney U test was used to compare continuous variables, which were expressed as means and standard deviations. Comparisons of categorical variables among LoY and Y‐preserved cancers groups were performed using the *χ* ^2^ (Pearson's Chi‐square) tests and Fisher's exact tests. The survival curves were estimated by Kaplan–Meier analysis, and *p* values were calculated by the log‐rank test. The prognostic impact of factors on patients' survival (PFS and OS) was evaluated by univariable and multivariable analysis with the Cox proportional hazards regression models. *p* < 0.05 was considered statistically significant. All statistical analyses were performed using R Studio Software.

## 3 Results

### 3.1 Patient Characteristics

Among all mCRC patients managed at Bordeaux University Hospital during the study period, we included those with available *KRAS/BRAF* mutational status assessed at the Tumor Biology and Tumor Bank Department (*n* = 170). Patients with a low tumor cell content (< 50%; *n* = 78) or with unavailable DNA (*n* = 1) were excluded. The flowchart of patient selection is presented in Figure S1.

Ninety‐one male patients with mCRC were finally included in the study. The mean age was 66.6 ± 12.7 years for the entire cohort. The primary tumor site was the rectum (*n* = 35, 41%), followed by the right and left colon, each accounting for 26% (*n* = 23) of cases. Among the included patients, 55% (*n* = 50) were diagnosed with synchronous mCRC, while 45% (*n* = 41) developed metastases after the initial diagnosis (median time = 17 months). Most patients had either one (*n* = 41, 45%) or two (*n* = 43, 47%) metastatic sites, with liver metastases observed in 67% (*n* = 60) of patients. Regarding genetic characteristics, 45% (*n* = 41) of patients carried an oncogenic *KRAS* mutation, 14% (*n* = 13) an oncogenic *BRAF* mutation, while 9% (*n* = 8) had MSI‐positive tumors. Thirty‐seven (44.6%) patients received chemotherapy without an EGFR inhibitor, mainly including FOLFOX (oxaliplatin, leucovorin, and 5‐fluorouracil), FOLFIRI (irinotecan, leucovorin, and 5‐fluorouracil), or FOLFIRINOX (oxaliplatin, irinotecan, leucovorin, and 5‐fluorouracil). For 43 patients (51.8%), these regimens were combined with targeted therapies against the EGFR receptor (cetuximab or panitumumab). Two patients (2.4%) received chemotherapy combined with a VEGF inhibitor (aflibercept or bevacizumab), and 1 (1.2%) received immunotherapy (Nivolumab + Ipilimumab). Treatment details were not specified for 8 patients. Complete descriptive statistics of quantitative variables are summarized in Table 1.

**TABLE 1:** Descriptive statistics of quantitative clinicopathological features.

All patients (*n* = 91)	
Clinicopathological features	Mean ± SD
Age	66.6 ± 12.7
BMI	26.2 ± 5.0
CEA (ng/mL)	89.1 ± 332.0
CA 19.9 (UI/mL)	3544 ± 14 848

	No of patients (%)
Localization	
Right colon	23 (26.4)
Transverse colon	6 (6.9)
Left colon	23 (26.4)
Rectum	35 (40.2)
Not specified	4
Number of metastatic sites	
1	41 (45.1)
2	43 (47.3)
3	5 (5.5)
4	2 (2.2)
Liver metastases	
No	30 (33.0)
Yes	60 (67.0)
Lymph node invasion	
No	68 (74.7)
Yes	23 (25.3)
OMS status	
0	46 (55.4)
1	31 (37.3)
2	5 (6.0)
3	1 (1.2)
Not specified	7
Mutation *KRAS*	
No	50 (54.9)
Yes	41 (45.1)
Mutation *BRAF*	
No	78 (85.7)
Yes	13 (14.3)
MSI status	
MSS	81 (91.0)
MSI	8 (9.0)
Not specified	2
LoY status	
LoY	46 (50.5)
Y preserved	45 (49.5)
Treatment	
Chemotherapy without EGFR inhibitor	37 (44.6)
Chemotherapy with EGFR inhibitor	43 (51.8)
Chemotherapy with VEGF inhibitor	2 (2.4)
Immunotherapy	1 (1.2)
Not specified	8
Treatment protocol	
FOLFOX without EGFR inhibitor	21 (25.3)
FOLFOX with EGFR inhibitor	13 (15.7)
FOLFIRI without EGFR inhibitor	2 (2.4)
FOLFIRI with EGFR inhibitor	15 (18.1)
FOLFIRI with VEGF inhibitor	2 (2.4)
FOLFIRINOX without EGFR inhibitor	11 (13.3)
FOLFIRINOX with EGFR inhibitor	9 (10.8)
Other without EGFR inhibitor	4 (4.8)
Other with EGFR inhibitor	6 (7.2)
Not specified	8

*Note:* ACE and CA 19.9 assays were performed at the time of metastasic stage diagnosis.

Abbreviations: MSI: microsatellite instability; MSS: microsatellite stability; SD: standard deviation.

### 3.2 Patients With LoY More Frequently Present With a Primary Rectal Tumor Than Those Without LoY

A total or partial LoY was detected in tumor cells for half of the patients (*n* = 46, 50.5%). We did not observe any difference in age between patients with LoY and Y‐preserved (67 ± 12.6 years VS 65 ± 12.8 years, respectively, *p* = 0.488, Table 2). There were no significant differences in other patients' characteristics between the 2 groups, including treatment adaptations or chemotherapy protocols (Table S2), except for primary tumor localization, with patients with LoY more frequently presenting with a rectal primary tumor compared with those with preserved Y (*p* = 0.038, Table 3). In particular, we did not notice any association between LoY and MSI, *BRAF*, or *KRAS* mutation status. Regarding rectal primary tumors (*n* = 35), LoY analysis was performed on DNA extracted from biopsies in 49% of patients (*n* = 17) and from surgical specimens in the remaining cases. Among the patients whose analysis was based on surgical specimens, 11 (61%) had received neoadjuvant treatment. Since this treatment could potentially influence LoY status compared with untreated tumors, we compared the proportion of LoY between treated and untreated tumors and found no significant difference (*p* = 0.596; Table S3), supporting the finding that rectal primary tumors more frequently harbor LoY than colon tumors.

**TABLE 2:** Association of LoY with patients' quantitative clinicopathological features.

All patients (*n* = 91)			
Clinicopathological features	LoY (*n* = 46)	Y preserved (*n* = 45)	*p*
	Mean ± SD	Mean ± SD	
Age (years)	67.5 ± 12.6	65.6 ± 12.8	0.488
BMI (kg/m^2^)	26.1 ± 5.1	26.4 ± 4.92	0.984
CEA (ng/mL)	130.9 ± 453.4	45.0 ± 94.1	0.459
CA 19.9 (UI/mL)	5037.5 ± 18978.1	3328.5 ± 12586.8	0.367

Abbreviation: SD: standard deviation.

**TABLE 3:** Association of LoY with clinicopathological features.

Clinicopathological features	LoY (*n* = 46) no of patients (%)	Y preserved (*n* = 45) no of patients (%)	*p*
Localization			
Right colon	7 (15.6)	16 (38.1)	0.038*
Transverse colon	5 (11.1)	1 (2.4)	
Left colon	11 (24.4)	12 (28.6)	
Rectum	22 (48.9)	13 (31)	
Not specified	1	3	
Number of metastatic sites			
1	20 (43.5)	21 (46.7)	0.936
2	23 (50.0)	20 (44.4)	
3	2 (4.3)	3 (6.7)	
4	1 (2.2)	1 (2.2)	
Liver metastases			
No	16 (34.8)	14 (31.1)	0.71
Yes	30 (65.2)	31 (68.9)	
Lymph node invasion			
No	36 (78.3)	32 (71.1)	0.433
Yes	10 (21.7)	13 (28.9)	
OMS status			
0	22 (52.4)	24 (58.5)	0.216
1	19 (45.2)	12 (29.3)	
2	1 (2.4)	4 (9.8)	
3	0 (0)	1 (2.4)	
Not specified	4	4	
Mutation *KRAS*			
No	27 (58.7)	23 (51.1)	0.467
Yes	19 (41.3)	22 (48.9)	
Mutation *BRAF*			
No	41 (89.1)	37 (82.2)	0.346
Yes	5 (10.9)	8 (17.8)	
MSI status			
MSS	43 (95.6)	38 (86.4)	0.13
MSI	2 (4.4)	6 (13.6)	
Not specified	1	1	

To explore whether the enrichment of LoY in rectal tumors could be observed in an external dataset, we analyzed male TCGA‐COAD and TCGA‐READ primary tumors with available unmasked chromosome Y copy‐number segment data. A total of 327 cases were evaluable, including 239 colon and 88 rectal cancers. Using the chromosome Y copy‐number score as a continuous variable, rectal tumors showed a slightly lower median chromosome Y signal than colon tumors, although this difference was not statistically significant (−0.965 [Q1–Q3: −1.450 to −0.696] vs. −0.940 [Q1–Q3: −1.290 to −0.600], *p* = 0.283). Similarly, rectal tumors were numerically more frequent among cases within the lowest chromosome Y quartile (26/88, 29.5% vs. 56/239, 23.4%; *p* = 0.314) and lowest chromosome Y decile (11/88, 12.5% vs. 22/239, 9.2%; Fisher *p* = 0.409), but these differences were not statistically significant (Table S4).

### 3.3 Outcomes: Loss of the Y Chromosome Is Associated With Improved Survival in Metastatic Colorectal Cancer

The median duration of PFS and OS were respectively 17 months (95% CI: 14–24) and 32 months (95% CI: 26—NR) (Figure S2) in the whole cohort. We next evaluated the impact of tumor genetics features (*KRAS* mutation and LoY status) on patients' survival (OS and PFS). First, we did not observe any significant impact of *KRAS* mutation status on patients' PFS (*KRAS* wild‐type: 18 months [95% CI, 14–27] vs. *KRAS* mutated: 16 months [95% CI, 11–28]; HR: 1.04, *p = 0.87*). Similarly, overall survival was comparable between the two groups (*KRAS* wild‐type: NR vs. *KRAS* mutated: 28 months [95% CI, 24—NR]; HR: 1.44, *p* = 0.22) (Figures S3A and S3B).

**FIGURE 1:**
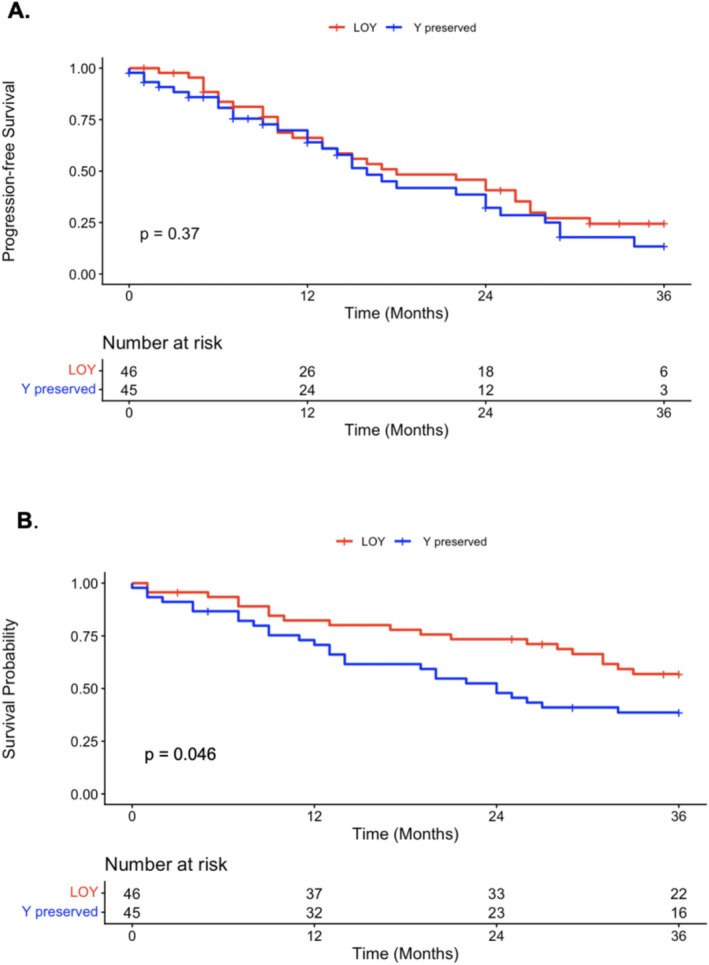
Patient's survival according to LoY status. (A) Kaplan–Meier Progression‐Free Survival (PFS) analysis according to LoY (red line) vs. preserved Y chromosome (blue line) status. (B) Kaplan–Meier overall survival (OS) analysis according to LoY (red line) vs. preserved Y chromosome (blue line) status.

Regarding LoY status, no significant difference was found in PFS between LoY tumors and tumors with a preserved Y chromosome, with median times to progression of 18 months [95% CI, 13–27] and 16 months [95% CI, 13–25], respectively (*p* = 0.37; Figure 1A). However, for OS, median survival times were NA [95% CI, 31—NA] versus 24 months [95% CI, 14—NA] for the LoY and preserved Y groups, respectively, and patients with LoY tumors showed a significantly better OS over the 3‐year follow‐up period (*p* = 0.046; Figure 1B).

Moreover, LoY remained significantly associated with improved survival after adjustment for age (HR = 0.48, 95% CI 0.24–0.94, *p* = 0.033), treatment adaptation (HR = 0.46, 95% CI 0.23–0.93, *p* = 0.030), and treatment type (HR = 0.49, 95% CI 0.25–0.97, *p* = 0.040). However, the association no longer reached statistical significance after adjustment for primary tumor localization (HR = 0.51, 95% CI 0.25–1.01, *p* = 0.054) or *KRAS* mutation status (HR = 0.51, 95% CI 0.26–1.02, *p* = 0.058). In a multivariable Cox proportional hazards model adjusting for age, tumor localization, treatment type, and *KRAS* mutation status, LoY was associated with a non‐significant trend toward reduced risk of death (HR = 0.52, 95% CI 0.25–1.10, *p* = 0.07).

**FIGURE 2:**
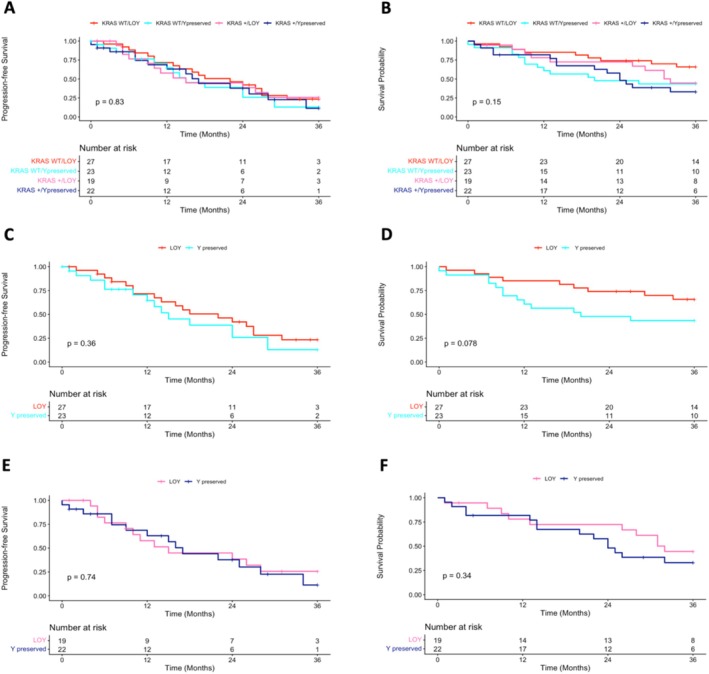
Patient's survival according to LoY and KRAS mutation status. Kaplan–Meier progression‐free survival (A) and overall survival (B) global analysis according to LoY status and *KRAS* mutation status. Kaplan–Meier progression‐free survival (C) and overall survival (D) analysis according to LoY in *KRAS* wild‐type tumors. Kaplan–Meier progression‐free survival (E) and overall survival (F) analysis according to LoY in *KRAS* mutated tumors.

Combining LoY and *KRAS* mutation status, no significant differences were observed in PFS or OS among the four patient subgroups (PFS *p* = 0.83; OS *p* = 0.15; Figure 2A,B). However, when assessing survival according to LoY status in *KRAS* wild‐type and *KRAS* mutated tumors separately, LoY‐positive patients showed numerically improved OS in the *KRAS* wild‐type subgroup, although this difference did not reach statistical significance (*p* = 0.078; Figure 2D,F). No differences were found in PFS between *KRAS* wild‐type tumors, nor in *KRAS*‐mutated tumors (Figure 2C,E). Finally, when analyzing survival according to LoY status and primary tumor localization, we did not observe any difference for PFS between LoY+ and Y‐preserved colon (LoY+: 17 months [95% CI, 10—NR] vs. Y‐preserved: 15 months [95% CI,10–29]; HR: 1.48, *p* = 0.26), and rectal tumors (LoY+: 24 months [95% CI, 13—NR] vs. Y‐preserved: 24 months [95% CI, 13—NR]; HR: 1.13, *p* = 0.79) (Figure S4A,C). However, a similar numerical trend toward better OS in LoY‐positive tumors was observed in both colon and rectal primary tumors, although these subgroup analyses did not reach statistical significance, likely due to the limited number of patients after stratification (Figure S4B,D).

We next explored the association between chromosome Y copy‐number signal and overall survival in TCGA male patients. In the combined TCGA‐COAD/READ cohort, patients within the lowest chromosome Y quartile had shorter overall survival than other patients (median OS: 1348 vs. 2532 days), with a significant association after adjustment for age and tumor location (HR = 1.99, 95% CI: 1.20–3.29, *p* = 0.00775; Table S5; Figure S5A). In TCGA‐COAD alone, lower chromosome Y signal remained associated with worse overall survival after adjustment for age, *KRAS* mutation status, and *BRAF* mutation status, both when analyzed as a continuous chromosome Y loss score (HR = 2.19, 95% CI: 1.41–3.41, *p* = 0.000482) and using the lowest chromosome Y quartile classification (median OS: 1348 vs. 2532 days; HR = 2.26, 95% CI: 1.23–4.16, *p* = 0.00902; Table S5; Figure S5B).

## 4 Discussion

The role and impact of LoY in tumor biology remain unclear. While some studies have identified LoY as a negative prognostic factor in certain cancers [12, 15], others have associated the activity of Y‐linked genes with more aggressive disease, especially in CRC [18]. Based on these findings, our study aimed to explore the association between LoY and the clinicopathological and molecular features of mCRC patients, as well as its prognostic value.

We observed in our cohort 50% of tumors exhibited LoY, which is consistent with the data in the literature. Indeed, recent publications report a wide range of LoY prevalence, from 25% to 75% (about 30% on average), and considerable variability across tumor types [14]. In CRC, LoY is reported in 30% to 50% of cases, though data often lack primary tumor exact anatomical location specificity [14]. In addition to LoY, we assessed the mutational status of key colorectal cancer oncogenes, such as *KRAS* and *BRAF*, along with MSI status, yielding frequencies comparable to those described in prior studies [19, 20].

Clinically, we observed an association between LoY and rectal primary tumor localization. LoY was significantly more frequent in rectal cancers (RC) than in colon cancers. This anatomical enrichment could reflect location‐specific biological or evolutionary features, as rectal and colon cancers are increasingly recognized as partially distinct entities [21]. Rectal cancers differ from colon cancers in terms of anatomical environment, vascular and lymphatic drainage, exposure to microbiota, and local microenvironmental constraints. These anatomical differences may shape specific immune, stromal, and inflammatory pressures [22], potentially influencing clonal selection and the emergence or maintenance of chromosomal alterations such as LoY. At the molecular level, tumor location is associated with differences in MSI frequency, chromosomal instability, immune infiltration, and patterns of clonal evolution. For instance, MSI is observed in approximately 15% of CRCs but is much less frequent in RC, where it ranges from 3% to 5% [23]. Furthermore, *KRAS* mutations appear to occur earlier and more clonally in right‐sided colon cancer, whereas RCs may show greater intratumoral heterogeneity [24]. Since LoY may occur in the context of broader chromosomal instability or clonal selection, these location‐specific molecular and microenvironmental features could contribute to its higher frequency in rectal tumors. However, this interpretation remains speculative, and larger cohorts integrating genome‐wide copy‐number alterations, *TP53* status, and tumor microenvironment characterization will be required to determine whether rectal tumors are genuinely predisposed to LoY.

To further explore this observation, we performed an exploratory analysis of male TCGA‐COAD and TCGA‐READ patients using unmasked copy‐number segment data. In this external dataset, rectal tumors showed a slightly lower chromosome Y copy‐number signal and were numerically more frequent among tumors with the lowest chromosome Y signal, but these differences did not reach statistical significance. Therefore, although TCGA showed a directionally concordant trend, it did not statistically validate the enrichment of LoY in rectal tumors observed in our institutional cohort. This discrepancy may reflect differences in cohort composition, tumor stage, tumor purity, and, importantly, assay methodology, as LoY was inferred indirectly from SNP‐array‐derived copy‐number segmentation in TCGA rather than measured using a dedicated chromosome Y‐specific ddPCR assay.

From a prognostic standpoint, our data showed an association between tumor LoY and better overall survival (OS) in mCRC. This association remained significant after adjustment for age, treatment type, and therapeutic adaptation, supporting the hypothesis that loss of Y‐linked pro‐tumoral genes may reduce tumor aggressiveness in specific contexts. In line with this, *Li et al.* identified *KDM5D*, a gene located on the Y chromosome, as having pro‐tumoral activity in CRC, with its loss potentially reducing tumor aggressiveness in the metastatic setting [18]. However, in our cohort, the association between LoY and OS disappeared after adjustment for primary tumor site and *KRAS* mutation status, as well as in multivariable analyses including *KRAS* status. This suggests that the prognostic association observed in our cohort may be partly influenced by the anatomical enrichment of LoY in rectal tumors and by molecular background.

To further assess the prognostic relevance of chromosome Y loss‐related signal, we performed exploratory survival analyses in TCGA. In contrast to our mCRC cohort, lower chromosome Y copy‐number signal in TCGA‐COAD/READ was associated with worse OS, including in TCGA‐COAD after adjustment for age and *KRAS*/*BRAF* mutation status. This apparent discrepancy should be interpreted with caution, as TCGA‐COAD/READ mainly includes primary tumors from a broader CRC population, whereas our cohort consisted exclusively of patients with mCRC. In addition, TCGA analyses relied on indirect inference from copy‐number segmentation, whereas LoY in our study was quantified by ddPCR. Differences in disease stage, cohort structure, treatment context, and assay methodology may therefore explain the divergent prognostic direction observed between the two datasets.

The relationship between LoY, *KRAS* status, and outcome also remains uncertain. In our cohort, analysis of OS according to LoY status in *KRAS* wild‐type and *KRAS*‐mutated tumors showed a non‐significant trend toward better prognosis in LoY‐positive tumors only within the *KRAS* wild‐type subgroup. This finding is intriguing, as Li et al. reported a pro‐aggressive role of *KDM5D* that was dependent on *KRAS* mutational activation. Our data therefore contrast with this observation and suggest that the relationship between LoY, *KRAS* status, and clinical outcome may be more complex than initially anticipated. Of note, in TCGA‐COAD, the adverse prognostic association of lower chromosome Y signal remained significant after adjustment for *KRAS* and *BRAF* status. However, individual *KRAS* variants may differ in their biological and clinical impact in CRC [17]. We could not reliably stratify *KRAS*‐mutated tumors in our cohort according to specific *KRAS* variants because *KRAS* status was assessed in routine clinical practice using different assays, including targeted multiplex testing for approximately half of the patients and NGS for the remaining cases. Future studies using homogeneous NGS‐based profiling will be required to determine whether specific *KRAS* alleles are differentially associated with LoY and clinical outcome.

Our results also contrast with the broader literature, in which chromosomal instability and consequently LoY is often associated with more advanced tumors and poorer prognosis. Indeed, genomic instability, often induced by inactivation of the tumor suppressor gene *TP53*, promotes multiple gains and losses of DNA copies [25]. LoY may simply be the result of a random loss of the Y chromosome, due to its small size and low gene density. Additionally, the rarity of genes it expresses outside the male reproductive system suggests limited selective pressure in favor of its preservation [26]. Tumors with frequent deleterious *TP53* mutations appear to be enriched in LoY in the Cancer Genome Atlas (TCGA) pan‐cancer cohort, an observation also valid in the Pan‐cancer analysis of whole genomes project database (PCAWG) [14]. More specifically, in CRC, LoY was more frequent in tumors with a *TP53* mutation, particularly in highly aneuploid tumors, highlighting a significant overlap between these two alterations. Consistent with the idea that chromosome Y loss may reflect broader chromosomal imbalance in at least a subset of CRCs, our exploratory TCGA analysis showed that lower chromosome Y copy‐number signal was associated with poorer OS, particularly in TCGA‐COAD. However, whether this association reflects a direct biological role of Y chromosome loss or merely captures a broader aneuploidy/chromosomal instability phenotype remains unresolved. Importantly, this observation does not apply uniformly to all tumors, as some cancers, such as uveal melanoma, show high fractions of LoY in the absence of frequent *TP53* alterations [14]. This suggests that, in these cancers, LoY is not a direct or specific consequence of genomic instability. Additionally, mosaic LoY in leukocytes is recognized as a genetic event associated with aging in men [27]. However, few data are available regarding the frequency of tumor LoY in relation to patient age, with some studies reporting a positive correlation [28, 29], while others have failed to reproduce this association, suggesting that the relationship may not be consistent across tumor types [14]. In our cohort, we did not observe a significant difference in age between patients with LoY‐positive and LoY‐negative tumors. These findings suggest that the occurrence of LoY in tumors may not be primarily driven by aging, but rather by the intrinsic characteristics of the tumor.

Regarding treatment response, we did not observe any significant difference in PFS between LoY+ and LoY‐ tumors. This suggests that LoY status does not predict first‐line treatment response but appears as a more global prognostic marker. In the overall cohort, heterogeneity in first‐line treatments may complicate the interpretation of the results, although no significant differences were observed between patients with and without LoY.

From a broader perspective, several studies have identified tumor LoY as a factor influencing microenvironment immune cell function, particularly regulatory T cells within the tumor microenvironment, and potentially affecting the response to immunotherapy [30]. *Li et al.* described the Y chromosome gene *KDM5D* as a promoter of immune escape in CRC, particularly through the downregulation of the MHC class I pathway [18]. In the same way, bladder cancer cells with LoY impair T cell function, promoting their exhaustion and sensitizing them to PD‐1‐targeted immunotherapy in vitro [15]. Finally, a recent pan‐cancer analysis revealed that LoY occurs not only in malignant epithelial cells but also in tumor‐infiltrating immune cells, particularly CD4+ and CD8+ T cells, where it was associated with immunosuppressive transcriptomic profiles and poorer clinical outcomes [31]. These findings support a potential mechanistic link between LoY observed in peripheral blood mononuclear cells and increased cancer mortality, highlighting LoY as a biomarker of prognostic relevance in male cancer patients. Interestingly, data from this study, including findings from in vivo models, suggest that LoY in immune cells may be induced by cancer cells themselves, prompting the authors to describe it as a potentially ‘contagious’ genetic event. In this context, investigating the impact of LoY on CRC immunotherapy response could be of particular interest, especially in MSI tumors, which were underrepresented in our study. However, LoY may occur less frequently within MSI‐positive tumors than in tumors characterized by chromosomal instability. Our results do not highlight any statistical link between MSI status and LoY. A larger MSI patient cohort would be needed to explore this hypothesis.

Finally, several limitations should be acknowledged. In addition to the limited cohort size, absence of specific *KRAS* variant annotations, and retrospective monocentric design, which restrict statistical power especially for adjusted and subgroup analyses, the exploratory TCGA analyses also have specific limitations. Chromosome Y loss was inferred from unmasked SNP‐array‐derived copy‐number segment data and not measured using a dedicated quantitative assay. In addition, TCGA‐COAD/READ differs substantially from our cohort in terms of disease stage, treatment context, and clinical composition, limiting direct comparison with our mCRC cohort. Our study also did not allow us to assess the spatial heterogeneity of LoY within individual tumors. LoY was assessed by ddPCR from a single selected tumor area, providing a robust quantitative evaluation in the analyzed sample but not capturing potential regional or subclonal heterogeneity. Moreover, LoY was assessed in primary tumors only, and matched metastatic lesions were not available. Therefore, we could not evaluate potential intertumoral heterogeneity between primary and metastatic sites or determine whether LoY represents an early stable chromosomal alteration retained during metastatic dissemination or a dynamic event acquired or selected during disease progression. Future studies comparing primary tumors and matched metastases, ideally using multi‐region sampling or single‐cell approaches, would be informative to clarify the timing, clonal architecture, and biological significance of LoY in colorectal cancer.

## 5 Conclusion

In conclusion, our results suggest that LoY is frequent in colorectal primary tumors and more frequent in rectal than in colon cancers. In our mCRC cohort, LoY was associated with improved overall survival, while a non‐significant trend toward improved OS was observed in *KRAS* wild‐type tumors. Exploratory TCGA analyses did not statistically confirm the enrichment of chromosome Y loss‐related signal in rectal tumors, but suggested that lower chromosome Y copy‐number signal may carry prognostic information in CRC, particularly in TCGA‐COAD. Expanding these analyses to larger cohorts and integrating data on chromosomal instability, *TP53* status, *KRAS* variants, and matched primary‐metastatic samples could help clarify whether LoY represents a stochastic event or a selected molecular feature in mCRC, with potential implications as a biomarker.

## Author Contributions

**A.I**.: conceptualization (supporting), investigation (lead), visualization (lead), writing – original draft (supporting), writing – review and editing (supporting). **M.D**., **B.F**., **D.S**.: investigation (supporting), writing – review and editing (supporting). **P.D**., **S.D**.: writing – review and editing (supporting). **O.M**.: conceptualization (supporting), supervision (supporting), writing – review and editing (supporting). **S.A**.: conceptualization (lead), supervision (lead), writing – original draft (lead), writing – review and editing (lead).

## Funding

This work received no specific grant from any funding agency in the public, commercial, or not‐for‐profit sectors but was supported by Bordeaux University Hospital.

## Ethics Statement

This study was conducted in accordance with the principles of the Declaration of Helsinki and local regulatory requirements. The study was approved by the Bordeaux University Hospital ethics review board on January 14, 2025 (reference CER‐BDX 2025–005). Clinical and biological data were collected retrospectively from medical records and analyzed in a pseudonymized manner. Archival tumor samples obtained during routine clinical care were used for molecular analyses in compliance with the ethics approval and applicable institutional requirements.

## Conflicts of Interest

The authors declare no conflicts of interest.

## Supporting information


**Table S1:** Primers and probes designed for the droplet digital PCR assay.
**Table S2:** Association of LoY with therapeutic features.
**Table S3:** Sample origin and neoadjuvant treatment of rectal primary tumors.
**Table S4:** Exploratory analysis of chromosome Y copy‐number signal in male TCGA‐COAD and TCGA‐READ primary tumors.
**Table S5:** Exploratory TCGA survival analyses according to chromosome Y copy‐number signal.
**Figure S1:** Patient's selection flowchart.
**Figure S2:** Global cohort patient's survival (A) Kaplan–Meier Progression‐Free Survival (PFS) analysis according to global cohort. (B) Kaplan–Meier overall survival (OS) analysis according to global cohort.
**Figure S3:** Patient's survival according to KRAS mutation status. (A) Kaplan–Meier Progression‐Free Survival (PFS) analysis according to KRAS mutated (red line) vs. KRAS wild‐type (blue line) status. (B) Kaplan–Meier overall survival (OS) according to KRAS mutated (red line) vs. KRAS wild‐type (blue line) status.
**Figure S4:** Patient's survival according to LoY and primary tumor localization. Kaplan–Meier progression‐free survival (A) and overall survival (B) according to LoY status in primary colon cancer tumors. Kaplan–Meier progression‐free survival (C) and overall survival (D) according to LoY status in primary rectal cancer tumors.
**Figure S5:** Patient survival according to chromosome Y copy‐number signal in TCGA datasets. Kaplan–Meier overall survival according to chromosome Y copy‐number signal in the combined TCGA‐COAD/READ cohort (A) and in the TCGA‐COAD cohort (B). Patients were classified as low chromosome Y signal when their chromosome Y copy‐number score was within the lowest quartile; all other patients were classified as higher chromosome Y signal.

## Data Availability

The data that support the findings of this study are available on request from the corresponding author, A.S.
