# Supplementary material for: Primary Tumor‐Associated Loss of the Y Chromosome and Clinical Outcome in Metastatic Colorectal Cancer
Source: Genes Chromosomes Cancer. 2026 Jun 14;65(6):e70147. doi: 10.1002/gcc.70147 (PMC13265838; doi:10.1002/gcc.70147)

**Supplementary data :**

**Supplemental Table 1.** *Primers and probes designed for the droplet digital PCR assay.*

| **Primer or probe name** | **Sequence** |
| --- | --- |
| Primer-amel-Fwd | 5’- *CCCCTGGGCACTGTAAAGAAT* |
| Primer-amel-Rev | 5’- *CCAAGCATCAGAGCTTAAACTG* |
| Probe-amelX | 5’- HEX-*CCAAATAAAGTGGTTTCTCAAGT*-BHQ |
| Probe-amelY | 5’- FAM-*CTTGAGAAACATCTGGGATAAAG*-BHQ. |

**Supplemental Table 2.** *Association of LoY with therapeutic features.*

| **Therapeutic features** | **LoY (n=46)** | **Y preserved (n=45)** |  |
| --- | --- | --- | --- |
|  | **No of patients (%)** | **No of patients (%)** | ***P value*** |
| **Treatment** |  |  |  |
| Chemotherapy without EGFR inhibitor | 17 (48.6) | 17 (44.7) | 0.920 |
| Chemotherapy with EGFR inhibitor | 18 (51.4) | 21 (55.3) |  |
| Not specified/Other | 11 | 7 |  |
| **Treatment protocol** |  |  |  |
| FOLFOX without EGFR inhibitor | 9 (22.0) | 12 (28.6) | 0.790 |
| FOLFOX with EGFR inhibitor | 7 (17.1) | 6 (14.3) |  |
| FOLFIRI without EGFR inhibitor | 1 (2.4) | 1 (2.4) |  |
| FOLFIRI with EGFR inhibitor | 6 (14.6) | 9 (21.4) |  |
| FOLFIRI with VEGF inhibitor | 0 (0) | 2 (4.8) |  |
| FOLFIRINOX without EGFR inhibitor | 7 (17.1) | 4 (9.5) |  |
| FOLFIRINOX with EGFR inhibitor | 5 (12.2) | 4 (9.5) |  |
| Other without EGFR inhibitor | 2 (4.9) | 2 (4.8) |  |
| Other with EGFR inhibitor | 4 (9.8) | 2 (4.8) |  |
| Not specified | 5 | 3 |  |
| **Therapeutic adaptation** |  |  |  |
| No | 16 (39) | 18 (48.6) | 0.392 |
| Yes | 25 (61) | 19 (51.4) |  |
| Other | 2 | 1 |  |
| Not specified | 5 | 7 |  |

**Supplemental Table 3.** *Sample origin and neoadjuvant treatment of rectal primary tumors*

| **RECTAL PRIMARY TUMOR PATIENTS (n=35)** | | | |
| --- | --- | --- | --- |
| **Sample type** | **LoY (n=22)** | **Y preserved (n=13)** |  |
|  | **No of patients (%)** | **No of patients (%)** | ***P value*** |
| **Biopsy** | **9** | **8** |  |
| **Resected tissues** | **13** | **5** |  |
| No neoadjuvant treatment | 6 (46.2) | 1 (20.0) | 0.596 |
| Neoadjuvant treatment | 7 (53.8) | 4 (80.0) |  |

**Supplemental Table 4.** *Exploratory analysis of chromosome Y copy-number signal in male TCGA-COAD and TCGA-READ primary tumors.*

| **Analysis** | **Colon**  **n=239** | **Rectum**  **n=88** | **Statistical test** | **p-value** |
| --- | --- | --- | --- | --- |
| Chromosome Y copy-number score, median [Q1–Q3] | −0.940  [−1.290 to −0.600] | −0.965  [−1.450 to −0.696] | Wilcoxon rank-sum test | 0.283 |
| Lowest chromosome Y quartile | 56/239 (23.4%) | 26/88 (29.5%) | Fisher’s exact test | 0.314 |
| Lowest chromosome Y decile | 22/239 (9.2%) | 11/88 (12.5%) | Fisher’s exact test | 0.409 |

TCGA, The Cancer Genome Atlas; COAD, colon adenocarcinoma; READ, rectum adenocarcinoma; chrY, chromosome Y; Q1, first quartile; Q3, third quartile.

**Supplemental Table 5.** *Exploratory TCGA survival analyses according to chromosome Y copy-number signal.*

| **Cohort** | **Analysis** | **Model** | **n (events)** | **HR** | **95% CI** | **p-value** | **PH assumption** |
| --- | --- | --- | --- | --- | --- | --- | --- |
| TCGA-COAD/READ | Lowest chromosome Y quartile | Adjusted for age and tumor location | 317 (66) | 1.99 | 1.20–3.29 | 0.00775 | Global p = 0.364 |
| TCGA-COAD | Chromosome Y loss score, continuous | Adjusted for age, KRAS and BRAF status | 231 (52) | 2.19 | 1.41–3.41 | 0.000482 | Global p = 0.83 |
| TCGA-COAD | Lowest chromosome Y quartile | Adjusted for age, KRAS and BRAF status | 231 (52) | 2.26 | 1.23–4.16 | 0.00902 | Global p = 0.91 |

TCGA, The Cancer Genome Atlas; COAD, colon adenocarcinoma; READ, rectum adenocarcinoma; chrY, chromosome Y; HR, hazard ratio; CI, confidence interval; PH, proportional hazards;

**Supplemental Figure 1.** *Patient’s selection flowchart*

*KRAS* mutation status determination at Bordeaux University Hospital Tumor Biology and Tumor Bank Department (n=170)

Male patients treated at the Bordeaux University Hospital for metastatic colon or rectal adenocarcinoma, initially or distantly, between January 2019 and December 2021

LoY analysis (n=91)

Excluded:
- Tumoral cellularity < 50% (n=78)

- Lack of DNA (n=1)

**
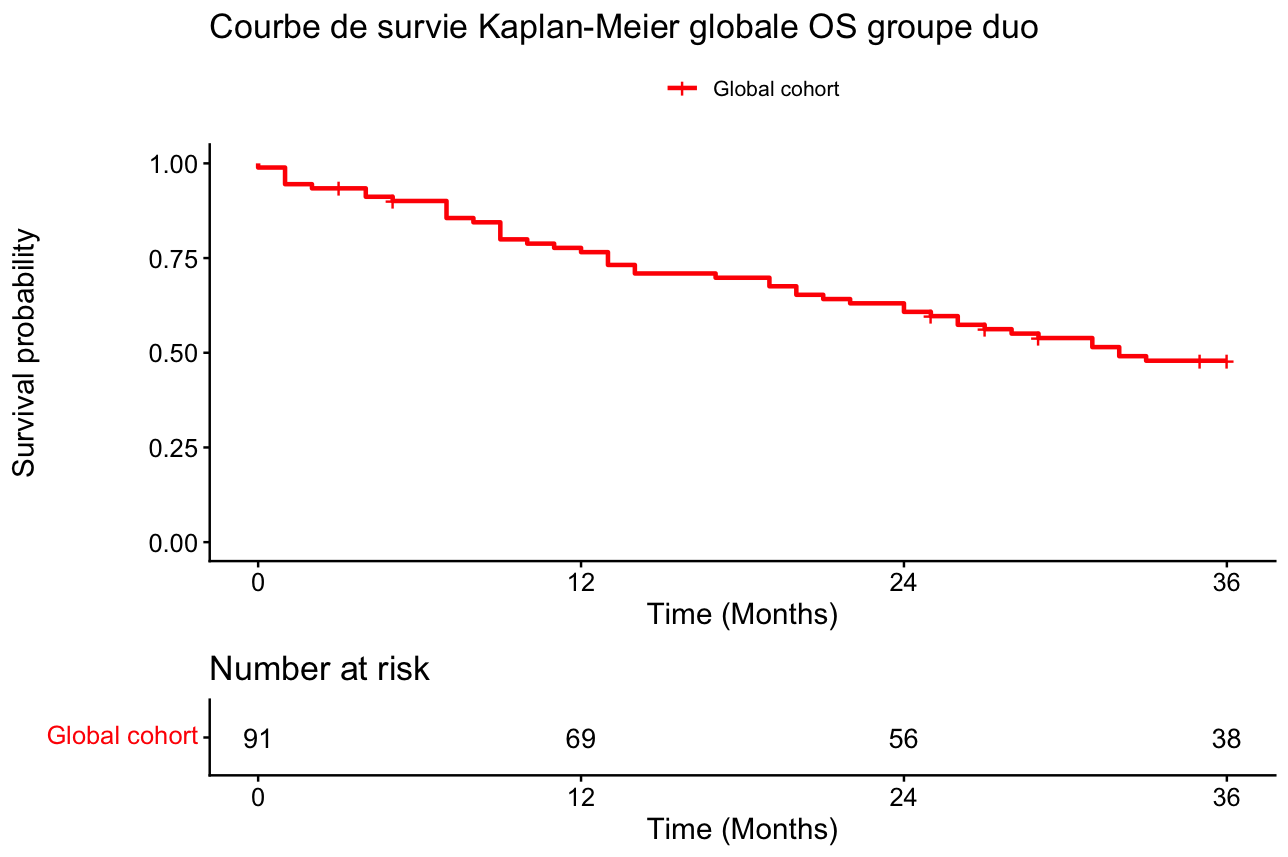

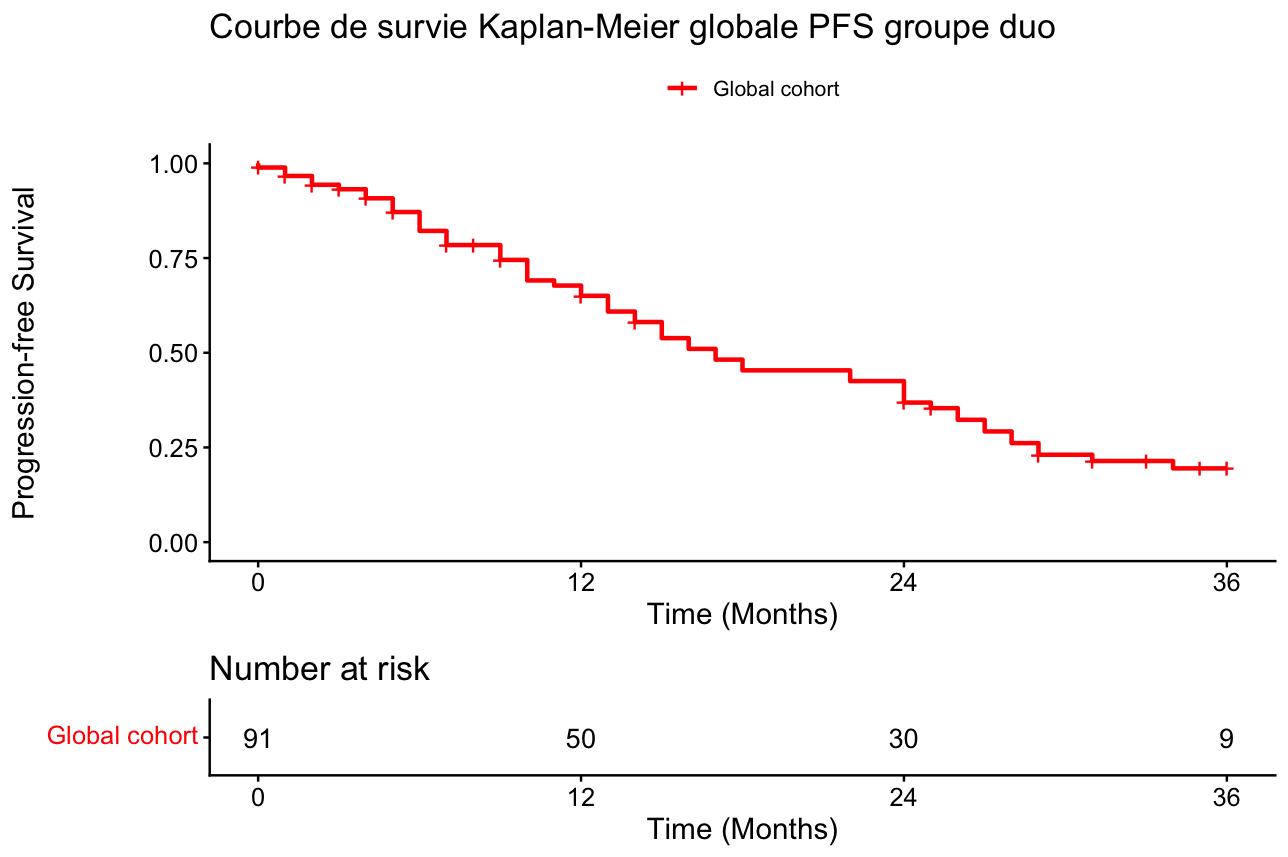
Supplemental Figure 2.** *Global cohort patient’s survival* **A**: Kaplan-Meier Progression-Free Survival (PFS) analysis according to global cohort**. B**: Kaplan-Meier overall survival (OS) analysis according to global cohort.

**A**.

**B**.

**Supplemental Figure 3.** *Patient’s survival according to KRAS mutation status*. **A**: Kaplan-Meier Progression-Free Survival (PFS) analysis according to *KRAS* mutated (red line) vs. *KRAS* wild-type (blue line) status. **B**: Kaplan-Meier overall survival (OS) according to *KRAS* mutated (red line) vs. *KRAS* wild-type (blue line) status.

**A**.


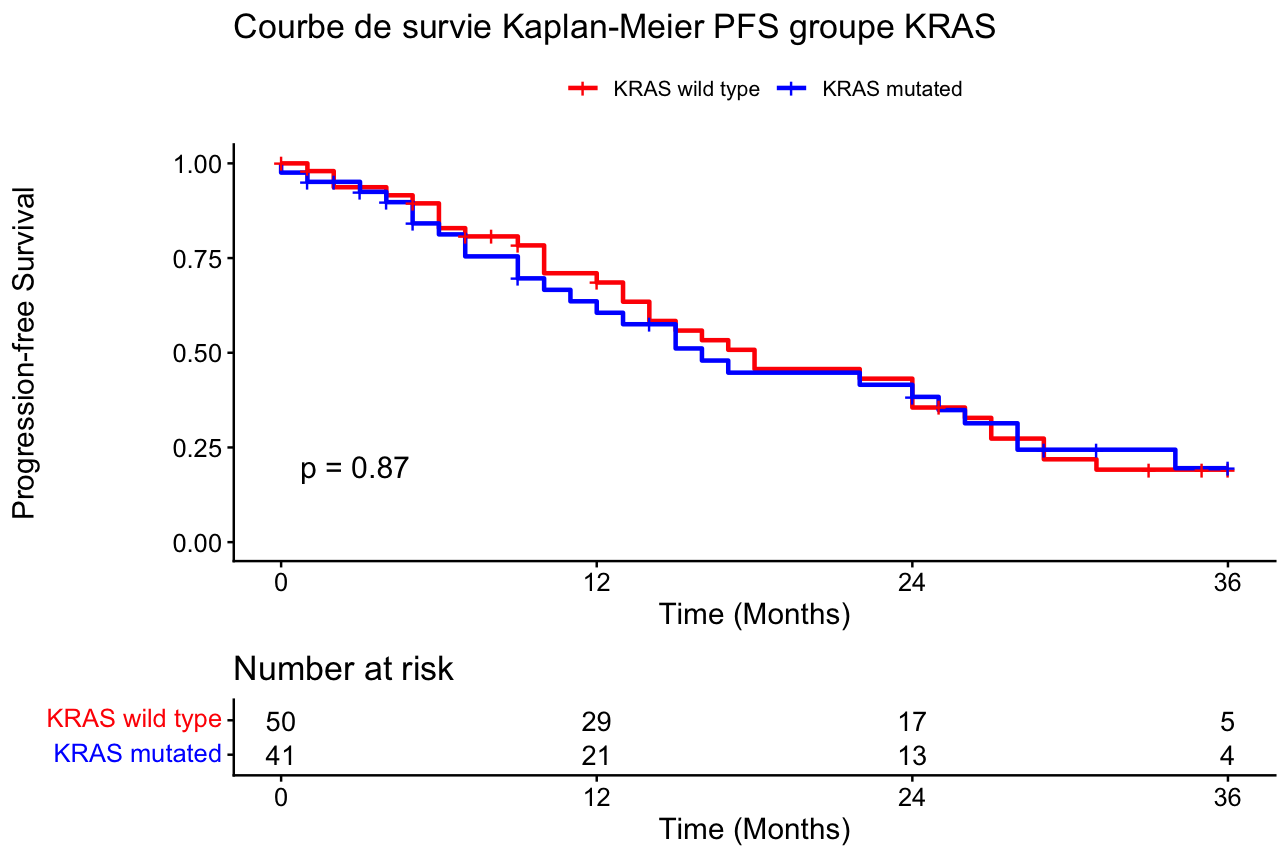


**
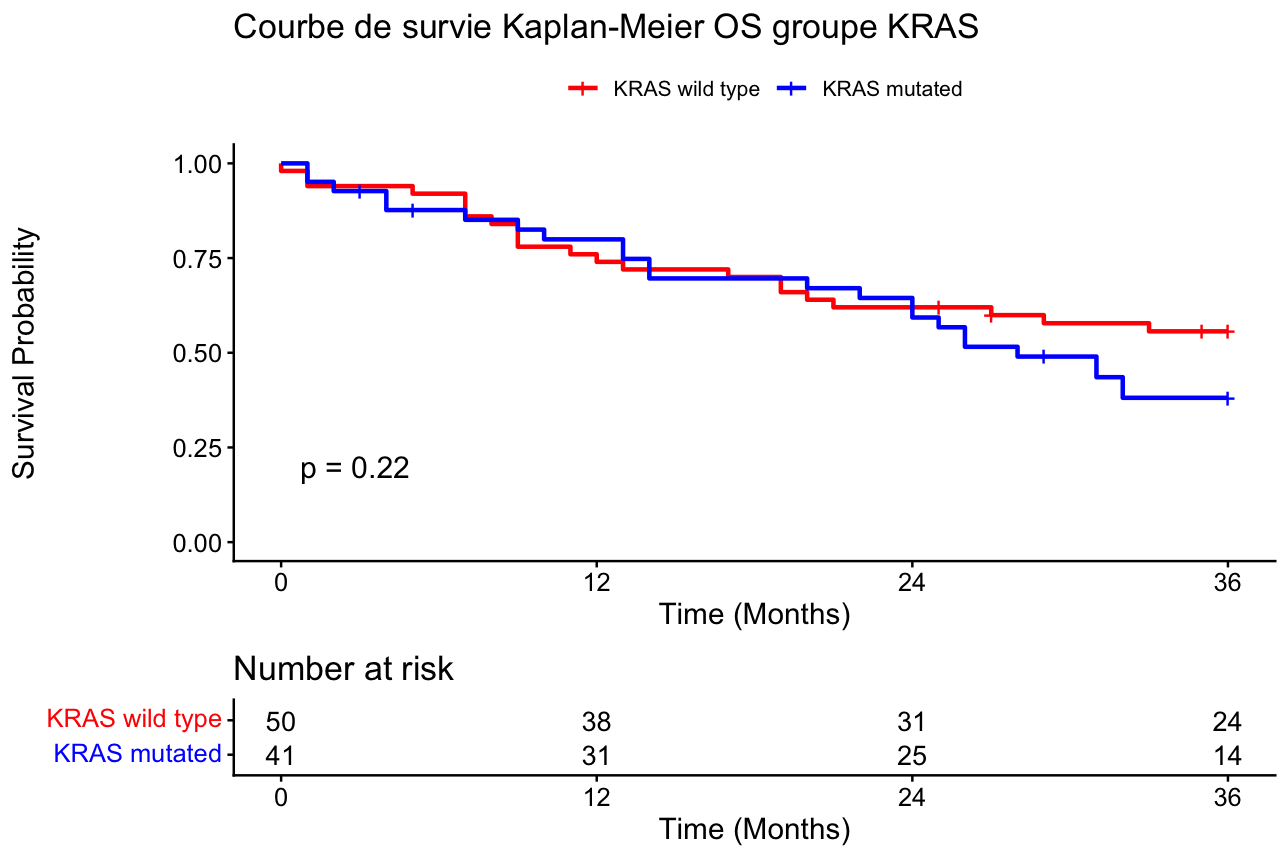
**

**B**.

**Supplemental Figure 4.** *Patient’s survival according to LoY and primary tumor localization*. Kaplan-Meier progression-free survival (**A**) and overall survival (**B**) according to LoY status in primary colon cancer tumors. Kaplan-Meier progression-free survival (**C**) and overall survival (**D**) according to LoY status in primary rectal **
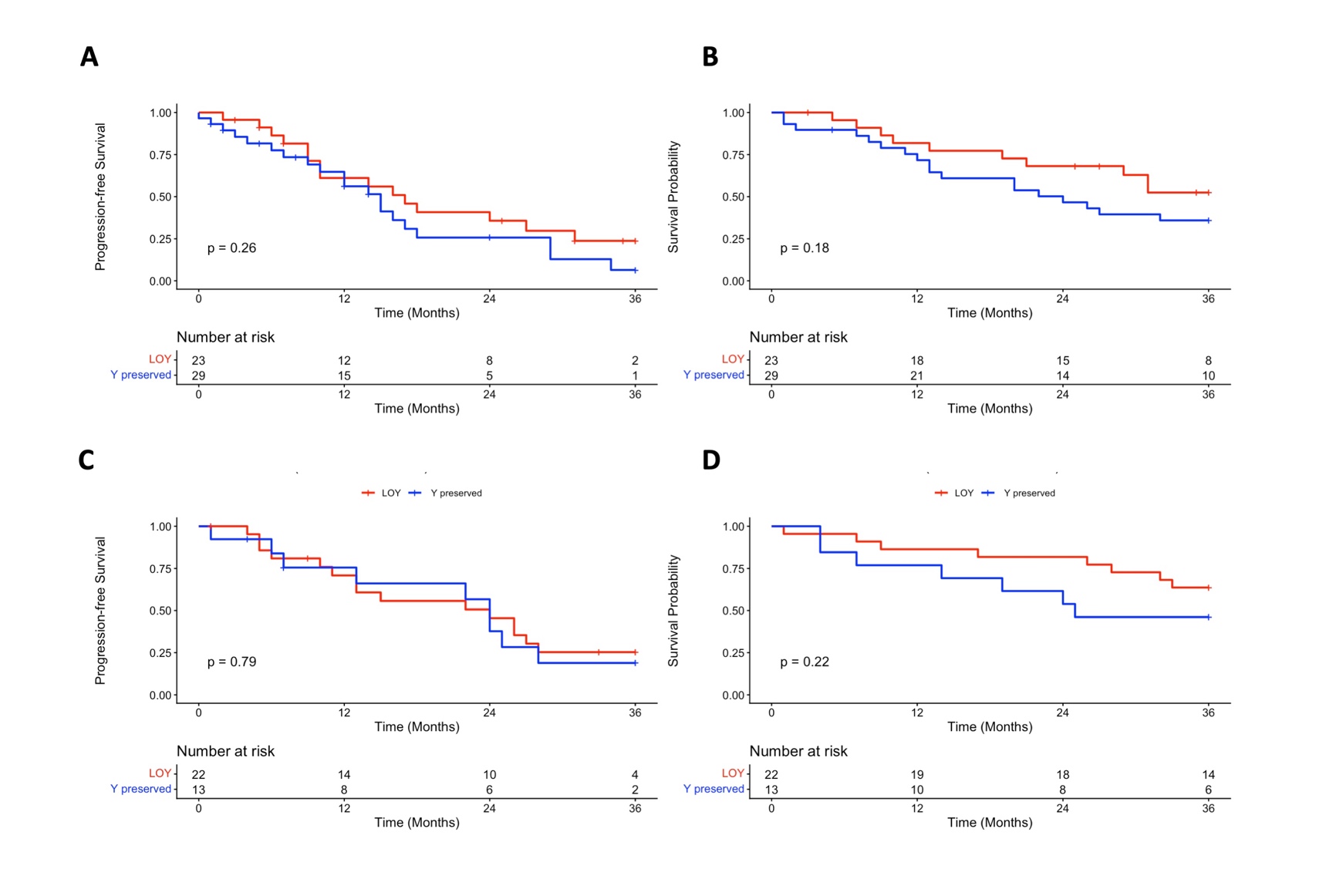
**cancer tumors.

**Supplemental Figure 5.** *Patient survival according to chromosome Y copy-number signal in TCGA datasets. Kaplan-Meier overall survival according to chromosome Y copy-number signal in the combined TCGA-COAD/READ cohort (****A****) and in the TCGA-COAD cohort (****B****). Patients were classified as low chromosome Y signal when their chromosome Y copy-number score was within the lowest quartile; all other patients were classified as higher chromosome Y signal.*


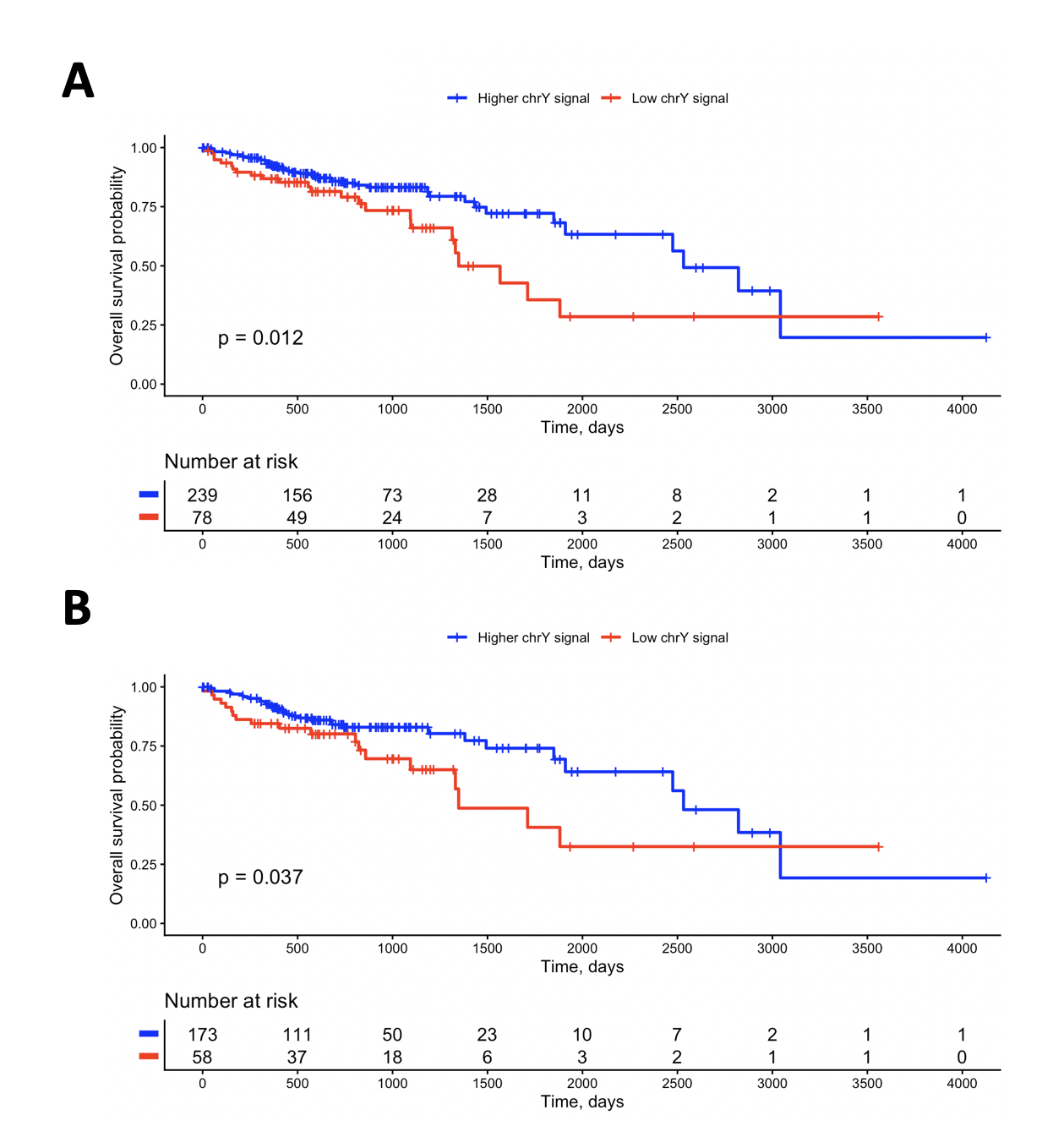

Supplement: Supplementary file 1 — Table S1: Primers and probes designed for the droplet digital PCR assay. Table S2: Association of LoY with therapeutic features. Table S3: Sample origin and neoadjuvant treatment of rectal primary tumors. Table S4: Exploratory analysis of chromosome Y copy‐number signal in male TCGA‐COAD and TCGA‐READ primary tumors. Table S5: Exploratory TCGA survival analyses according to chromosome Y copy‐number signal. Figure S1: Patient's selection flowchart. Figure S2: Global cohort patient's survival (A) Kaplan–Meier Progression‐Free Survival (PFS) analysis according to global cohort. (B) Kaplan–Meier overall survival (OS) analysis according to global cohort. Figure S3: Patient's survival according to KRAS mutation status. (A) Kaplan–Meier Progression‐Free Survival (PFS) analysis according to KRAS mutated (red line) vs. KRAS wild‐type (blue line) status. (B) Kaplan–Meier overall survival (OS) according to KRAS mutated (red line) vs. KRAS wild‐type (blue line) status. Figure S4: Patient's survival according to LoY and primary tumor localization. Kaplan–Meier progression‐free survival (A) and overall survival (B) according to LoY status in primary colon cancer tumors. Kaplan–Meier progression‐free survival (C) and overall survival (D) according to LoY status in primary rectal cancer tumors. Figure S5: Patient survival according to chromosome Y copy‐number signal in TCGA datasets. Kaplan–Meier overall survival according to chromosome Y copy‐number signal in the combined TCGA‐COAD/READ cohort (A) and in the TCGA‐COAD cohort (B). Patients were classified as low chromosome Y signal when their chromosome Y copy‐number score was within the lowest quartile; all other patients were classified as higher chromosome Y signal. [file GCC-65-e70147-s001.docx]
